# Harnessing the genetic potential of exotic sorghum germplasm for drought resilience in arid regions of Ethiopia

**DOI:** 10.3389/fpls.2025.1548591

**Published:** 2025-04-09

**Authors:** Techale Birhan, Nezif Abajebel, Misganu Wakjira, Tesfaye Mitiku, Vincent Vadez, Million Tadege, Andrew H. Paterson, Kassahun Bantte

**Affiliations:** ^1^ Institute for Agricultural Biosciences, Oklahoma State University, Ardmore, OK, United States; ^2^ Department of Horticulture and Plant Science, Jimma University, Jimma, Ethiopia; ^3^ Université de Montpellier, IRD, DIADE, Montpellier, France; ^4^ Centre d’Etude Régional pour l’Amélioration de l’Adaptation à la Sécheresse (CERAAS), Thiès, Senegal; ^5^ Plant Genome Mapping Laboratory, University of Georgia, Athens, GA, United States

**Keywords:** sorghum, BCNAM, drought stress, flowering time, transpiration efficiency, landraces

## Abstract

The narrow genetic diversity of modern sorghum varieties indicates that favorable alleles for the breeding process are frequently lacking in elite germplasm. To address this challenge, here, we use a multiparent breeding technique that employs exotic germplasm to introduce new alleles into an elite gene pool with the goal of identifying potential segregants that combine suitable yield and quality with drought resilience components. The genetic materials used consisted of 1,260 backcross-nested association mapping (BCNAM) BC1F4 lines from 13 populations developed by crossing 13 exotic accessions, earlier screened for diverse drought resilience traits, to a locally important elite cultivar (Teshale), also including the 14 parents. The populations (50 to 200 per family) were evaluated using an alpha lattice design at three locations representative of the major sorghum production regions in Ethiopia. Progenies displayed rich variability in most studied traits, with some outperforming existing varieties in most of these traits. Lines, such as 1180, 1373, 1318, and 1, gave the highest average grain yield, outperforming Teshale, the recurrent parent. Lines 1199, 1263, 1101, and 1204 had the shortest average days to flowering making them more suitable to escape moisture stress periods. Progenies originating from high transpiration efficiency (TE) donor parents showed higher grain yields, early flowering, and maturity, while those from donors with high water extraction showed low yields, delayed flowering, and maturity. In general, donor parents IS14556 and IS16044 with high TE seemed effective in conferring drought tolerance-related characters based on high average performance of all lines from these donors and higher frequencies of transgressants among their progenies. These carefully chosen crosses and the BCNAM approach show promise as an effective vehicle to transfer beneficial alleles from exotic sorghum germplasm into Ethiopian elite genetic backgrounds, in particular, toward improving adaptation of this essential staple crop to the severe droughts that endanger regional food security. These findings highlight that sorghum improvement in water-limited areas may profit from use of exotic genetic resources conferring traits, such as transpiration efficiency, coupled with selection for 1,000-seed weight, leaf senescence, plant height, and flowering time.

## Introduction

Sorghum [*Sorghum bicolor* (L.)] is the leading traditional food in Ethiopia comprising 19.5% of the total cereal production in the country (CSA, 2017). The crop is mainly cultivated in dry and hot lowlands under rainfed conditions where terminal drought stress is a common problem (Masresha et al., 2011). With a center of diversity also thought to be in or near Ethiopia and having evolved under warm and dry conditions, sorghum has higher tolerance to drought and high temperature stress than many other crops. Nonetheless, drought stress remains the major cause of sorghum yield reduction and food insecurity in Ethiopia where crop cultivation mainly depends on seasonal rains (Abdalla and Gamar, 2011). More than 80% of Ethiopia’s sorghum is grown in regions experiencing moderate-to-severe environmental stress (Gebre et al., 2014). This results in notable yield losses for the agroecosystem that depends upon rainwater. Unpredictable rainfall, high microclimatic variability within fields, and post flowering drought stress are common in the country (Abreha et al., 2022).

The success of new crop variety development is primarily contingent upon the availability of germplasm that exhibits desirable traits, as well as the implementation of effective screening methodologies to distinguish between genotypes (Saad et al., 2022). A broad genetic diversity is crucial, as it provides the essential gene pool for developing and producing new varieties with agriculturally important traits that are critical for addressing current and future challenges in agriculture. Exotic sorghum landraces have proven to be valuable sources of desirable traits in the development of crop varieties that are resilient to evolving disease pressures and changing climatic conditions. However, directly utilizing landraces in breeding programs presents certain limitations, particularly due to linkage drag. To overcome this challenge, converting landraces into more advanced breeding lines, such as BCNAM, will enhance their suitability for practical application and maximize their potential benefits. Available scientific evidence indicated that improved sorghum hybrids and varieties are less diverse than their landraces and wild types (Mace et al., 2013; Muraya et al., 2013). The low diversity of crop cultivars is mainly because most crop breeders focus on crossing “the best of the best,” using limited numbers of adapted and improved materials that have the most desirable traits and avoiding local and exotic landraces. Such a narrow genetic base of cultivars might increase crop vulnerability.

Given the predicted impact of climate change, population growth, and changing production, crops need to deliver increased yields under progressively more challenging conditions (Muraya et al., 2013), and the introduction of new alleles from exotic landraces is an important strategy to develop improved varieties. Multi-parental advanced breeding lines derived from intraspecific crosses are a powerful strategy to evaluate and transfer novel alleles from exotic progenitors into adapted genetic backgrounds (Jordan et al., 2011). Once alleles of interest have been transferred into a target elite background, a certain number of backcrosses may be needed to dilute unnecessary traits often associated with introgressions and restore the agronomic potential of elite parents. Over the past few decades, there have been successes in introducing traits from exotic species into cultivated crops mostly for overcoming biotic stresses (Mace et al., 2013). The past decade has seen the rise of BCNAM (backcross-nested association mapping) as a design offering great advantages for genetic studies in plants, integrating the mapping of quantitative trait loci and providing germplasm for breeding programs (Liu et al., 2003). It also allows one to evaluate a wide range of genes from diverse sources in the context of elite sorghum backgrounds that have the local adaptation and quality factors suitable for widespread use in target regions (Yu et al., 2006). It is an effective way to introduce new alleles from exotic germplasms into elite genetic backgrounds and to get immediately improved elite transgressive segregants for different traits through selection (Jordan et al., 2011; Chapman et al., 2003). Several studies are being conducted worldwide to take advantage of multiparent advanced generation-derived breeding lines (Melanie et al., 2018). Prior to this, Jordan et al. (2011) assessed the performance of 56 BC1F4 sorghum families and found some novel progenies that outperformed the recurrent parent in terms of grain yield and maturity. In a similar vein, Mace et al. (2013) and Buckler et al. (2009) assessed subsets of NAM populations from 24 families of sorghum and 25 families of maize, respectively, and found significant variations in flowering time. Numerous global studies have investigated drought tolerance in sorghum by examining various morphological traits and their association with drought tolerance (Haussmann et al., 2007; Shinde et al., 2010; Mahajan et al., 2011; Ali, 2012; Mohankumar et al., 2013; Kamatar et al., 2015). In addition, genotypes exhibiting promising responses to terminal drought stress have been identified.

Traits, such as transpiration efficiency (TE) and water extraction ability, are believed to contribute to drought stress resilience in sorghum. This makes those traits a potentially valuable trait for incorporation into breeding programs. For instance, research works showed that the sorghum germplasm exhibits significant genetic variation for transpiration efficiency (TE) (Hammer et al., 2006; Xin et al., 2008). Sorghum genotypes with high TE showed strong correlation with increased biomass accumulation (Raymundo et al., 2024). Other simulation studies also showed a substantial effect of increased TE on grain yield, particularly under drought stress (Hammer et al., 2006). According to Vadez et al. (2011a), TE and water extraction are not mutually exclusive and were the main drivers of yield differences in sorghum during terminal drought. Moreover, other traits are used to screen genotypes and evaluate stress tolerance. For instance, early flowering or maturity is commonly seen as a drought-escape strategy, and reduced flowering delay under drought has been proposed as an indicator of drought tolerance in some environments (Monkham et al., 2015). Leaf senescence and head exertion have been also targeted for phenotyping drought response in sorghum (Harris et al., 2007).

Nevertheless, there has been little progress in enhancing Ethiopian elite genetic backgrounds for drought tolerance by introducing favorable alleles from exotic sorghum germplasm. Of particular importance to Ethiopia, a wide range of variations in transpiration efficiency and water extraction capacity has been reported in ICRISAT’s sorghum collections (Vadez et al., 2011a, b). There, transpiration efficiency was measured at the plant level (biomass divided by cumulated transpiration) in robust assessments in lysimeters over the entire crop cycle (Vadez et al., 2024). Many of these genotypes are not suitable for cultivation in Ethiopia due to lack of adaptation to local conditions or local grain quality preferences, but they offer a broad spectrum of drought response mechanisms. Exotic alleles from these superior variants could be transferred to a locally suitable germplasm through a BCNAM approach.

Therefore, the main objective of this study was to transfer such traits, evaluate the performance of advanced backcross-derived populations, and identify potential segregants for yield and drought tolerance-related traits under moisture stress conditions.

## Materials and methods

### Plant genetic materials

Thirteen populations, collectively referred to as the backcross-nested association mapping set (BCNAM), were developed using an elite line (Teshale) with local adaptation as the recurrent parent and crossing it with a range of donor lines (as paternal parents) that are exotic to Ethiopian conditions. These donor lines were selected from a panel with a diverse spectrum of drought responsiveness traits such as high water extraction ability and transpiration efficiency (Vadez et al., 2011a, b). The 13 donor paternal lines (**Table 1**) were sourced from ICRISAT, and the BCNAM populations were developed at Jimma University College of Agriculture. The transpiration efficiency of the donor parents were measured at the plant level (biomass divided by cumulated transpiration) in robust assessments in lysimeters over the entire crop cycle (Vadez et al., 2024). The recurrent parent, *Teshale*, is an Ethiopian variety (caudatum type), which is highly preferred by Ethiopian farmers for its high grain yield and grain color but is relatively susceptible to moisture stress. The populations for this study were created using a nested design, where each subpopulation was developed by crossing the elite maternal parent (Teshale) with each one of the 13 lines, followed by backcrossing the F1 generation to generate BC1F1 populations. Subsequent selfing was carried out to produce BC1F4 populations using the single seed descent method. The final panel lodged in our gene bank and used for field evaluation consisted of 1,260 BC1F4 progenies (50–200 lines per family) from 13 backcross populations involving 13 exotic parents and one adapted Ethiopian elite line.

**Table 1:** Parental lines used for backcross-nested association mapping population development (BCNAM) and their targeted traits in relation to drought tolerance.

Genotype	Origin	Biological status	Source	Target traits
IS2205	India	Landrace	ICRISAT	H_2_O extraction
IS3583	Sudan	Landrace	ICRISAT	TE
IS9911	Sudan	Landrace	ICRISAT	HI
IS14556	Ethiopia	Landrace	ICRISAT	TE
IS16044	Cameroon	Landrace	ICRISAT	TE
IS16173	Cameroon	Landrace	ICRISAT	TE
IS22325	Botswana	Landrace	ICRISAT	TE
IS10876	Nigeria	Landrace	ICRISAT	TE
IS14298	South Africa	Landrace	ICRISAT	HI
IS14446	Sudan	Landrace	ICRISAT	H_2_O extraction
IS15428	Cameroon	Landrace	ICRISAT	TE
IS23988	Yemen	Landrace	ICRISAT	H_2_O extraction
IS32234	Yemen	Landrace	ICRISAT	HI
Teshale	Ethiopia	Improved variety	Melkassa	Recurrent parent

§Source information is obtained from the International Crops Research Institute for the Semi-Arid Tropics (ICRISAT): http://genebank.icrisat.org/IND/Passport?Crop=Sorghum.

### Field experiment sites, layout, and management

**Figure 1:**
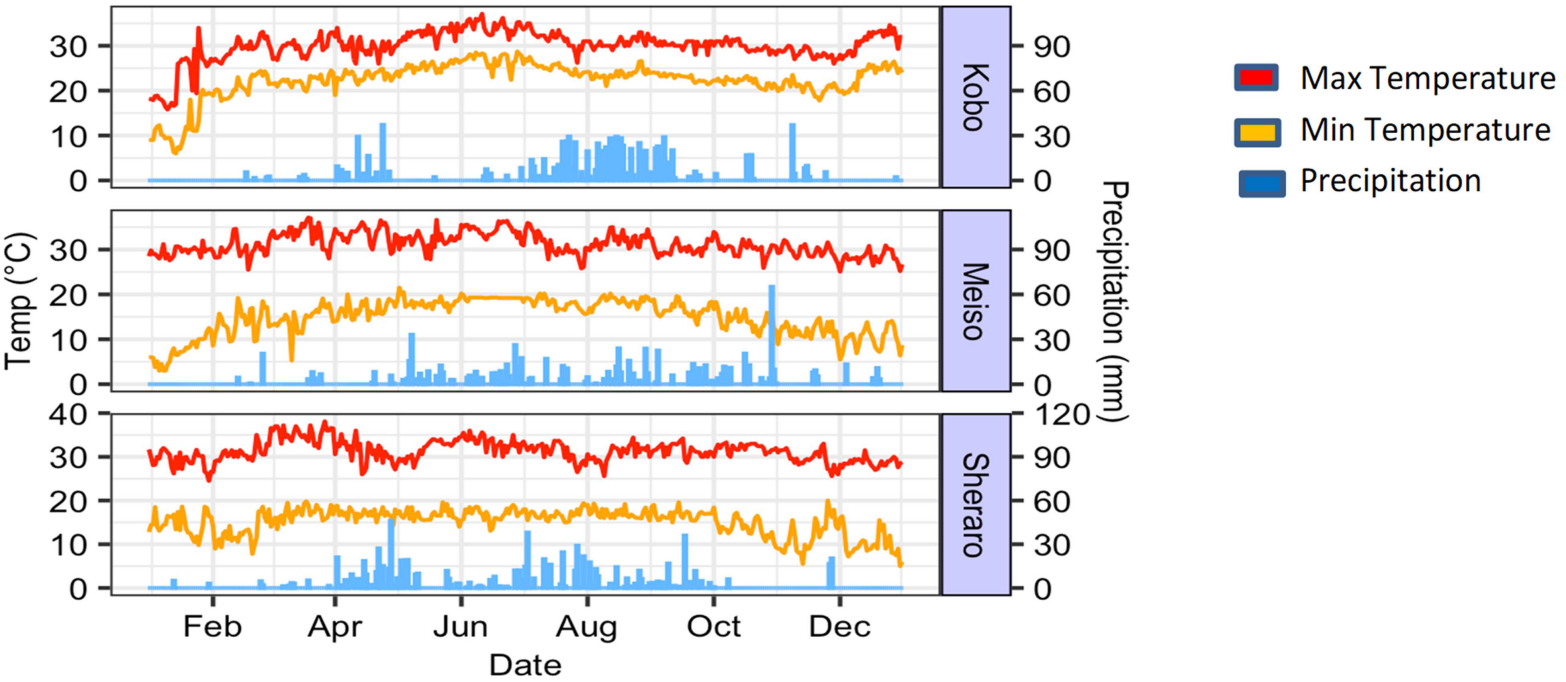
Weather conditions at the three field trial sites in Ethiopia (Kobo, Meiso and Sheraro).

The 1,260 sorghum lines and 14 parents were evaluated during the main cropping season in 2018 at three commonly used testing sites for moisture stress research: Kobo (12°09′N, 39°38′E), Meiso (09°14′N, 40°45′E), and Sheraro (14°23′N, 37°46′E). The field trial was carried out between July and December in all the three locations. The BCNAM population was evaluated under natural moisture stress conditions employing an alpha lattice design with two replications at each of the locations. The distance between rows was set at 75 cm, while the spacing between plants was 20 cm. In each plot, the seeds were sown in a single 4-m-long row, and planting was done manually followed by thinning to 20-cm space between plants. All the other agronomic practices were done following the standard procedures for sorghum. The sites are moisture-stressed areas in Ethiopia where post-flowering moisture stress is predominant, with warm to hot summers. No irrigation systems were available at the test sites. As a result, the populations were evaluated only under naturally occurring moisture stress conditions. We collected meteorological data, including daily precipitation, and minimum and maximum temperatures, from neighboring weather stations for the period covering the experiment seasons (**Figure 1**).

### Phenotypic measurements

Days to flowering (DTF) and days to maturity (DTM) were the number of days from emergence to flowering and physiological maturity, respectively. Plant height (PHT) was measured as the distance from the soil level to the tip of the main stem panicle. Grain yield per plant (GYP) was the total grain weight in grams of threshed panicles of one plant averaged from five randomly selected plants per plot. The number of tillers per plant (NT) and the number of leaves per plant (NL) were measured by counting five randomly chosen plants at maturity. Panicle exertion (HE) was measured between the bases of the flag leaf to the base of the panicle in centimeters and rated as 1 = slightly exserted (<2 cm but ligule of flag leaf definitively below inflorescence base), 2 = moderately exserted (2–10 cm between the ligule and inflorescence base), 3 = well exserted (>10 cm between ligule and the inflorescence base). Leaf senescence (LS) was recorded by visual ratings on a scale of 1 to 9 based on the degree of leaf death at physiological maturity on a plot basis as 1 = very slightly senescent (10%), 3 = slightly senescent (25%), 5 = intermediate (50%), 7 = mostly senescent (75%), and 9 = completely senescent (100%) based on sorghum descriptors (IBPGR, 1993).

### Data analysis

Restricted maximum likelihood (REML) was used to estimate variance components by using R software (version 4.4.1) to investigate statistical differences between the BC1F4 lines. To test the significance of the environment, family, genotype nested within family, family by environment interaction, and genotype nested within family by environment interaction, analysis of variance (ANOVA) was first performed across all three environments. Using a mixed linear model implemented in the lme4 package, the trait best linear unbiased predictions (BLUPs) were estimated for each line within each environment (Bates et al., 2015). Genotypic and phenotypic coefficients of variation and expected genetic advance were estimated according to Burton and Devane (1953) and Johnson et al. (1955), respectively. Simple correlation coefficients (r) were calculated among all traits based on the predicted means of the lines (Wright, 1968). Transgressive segregants were identified by finding the number of plants exceeding the mean value of the higher parent or lagging behind the mean value of the lower parent by critical difference at the 5% level (Shreya et al., 2017).


$$Y_{ijk}=\mu +F_{i}+G{\left(F\right)}_{ij}+R_{k}+\epsilon_{ijk}$$

where Y denotes the raw phenotypic data, F indicates the individual family within the BCNAM population, G(F) represents the genotype nested within the family, R stands for replication, and ϵ signifies random error. The Pearson correlation coefficients between traits were calculated using BLUPs. Broad-sense heritability was assessed as the ratio of total phenotypic variance accounted for by the combined family and line terms (Wallace et al., 2016).

## Results

### Rainfall pattern and soil characteristics

Clear drought stresses, including plant losses, were observed at all three environments during the cropping season, in particular with very little rainfall in the final 2 months of the growing seasons (**Figure 1**). While we aimed for a population of 20 plants per plot, we realized an average of 11.5 in Kobo, 9.0 in Meiso, and 13.87 plants in Sheraro, which could be attributed to the pattern and distribution of rainfall in the study areas. However, to check if stand count could have had an effect on the variation within our traits, we used simple regression analyses between these traits and plant counts and found non-significant small R-values at each of the three sites.

The cumulative rainfall before sowing (January to June) was 179.4 mm in Kobo and 298.24 mm in Meiso, with 423.32 mm in Sheraro being the highest rainfall. The total rainfall during the cropping period (July to November) was 420.5 mm in Kobo, 465.4 mm in Meiso, and 503.2 mm in Sheraro. It was highest from August to September in all three sites and lowest during October through December. Sherero had the most rainfall throughout the trial season of the three environments. According to the FAO’s soil classification system, the predominant soil type in Meiso is Vertic Cambisol, which has a clay texture. Most of the soils at the Kobo and Sheraro sites are Eutric Vertisols with a clay texture (FAO, 2007).

### Phenotypic trait variation and heritability

Analysis of variance (ANOVA) was performed across all three environments to determine the significance of the environment, family, genotype nested within family, family by environment interaction, and genotype nested within family by environment interaction (**Table 2**). Significant differences were found among lines for all traits except panicle harvest index and grain filling period. Analysis of variance also showed statistically significant differences between family, whereas the family nested within environment interaction mean squares were non-significant for most of the studied traits except grain filling period.

**Table 2:** Estimates of variance components for 11 traits of 1,260 sorghum BC1F4 genotypes grown at Kobo, Sheraro, and Meiso during the 2018 cropping season.

Traits	Genotype	Environment	Genotype × (environment)	Family	Family × (environment)	Residual
DTF	345.06**	10.53ns	12.36**	40.30*	2.45ns	0.27
DTM	401.13**	4.33ns	80.90*	3.04*	9.80ns	20.40
GFP	12.23ns	145.78**	28.59*	13.50*	4.52*	1.03
PHT	428.19**	3,687.00**	200.71*	11.30*	12.34ns	20.06
NT	32.71*	0.29ns	11.37ns	2.89ns	9.23ns	0.03
NL	45.08*	6.12ns	9.34**	2.50ns	3.00ns	0.08
LS	30.76**	0.06ns	14.40ns	10.83**	5.89ns	0.35
HE	112.06**	1.35*	25.36*	52.90*	1.17ns	1.27
GYP	280.02**	1.70ns	108.53*	102.20*	28.32ns	24.23
TSW	39.62**	2.90ns	29.00*	125.60*	1.75ns	2.18
PHI	34.92ns	13.2ns	95.30*	325.00*	35.00ns	30.33

**, *Significant at 1% and 5% probability levels, respectively, DTF, days to flowering; DTM, days to maturity; GFP, grain filling period; PHT, plant height; NT, number of tillers per plant; NL, number of leaves per plant; LS, leaf senescence; HE, panicle exertion; GYP, grain yield per panicle; TSW, 1,000-seed weight; PHI, panicle harvest index; ns, non-significant.

**Figure 2:**
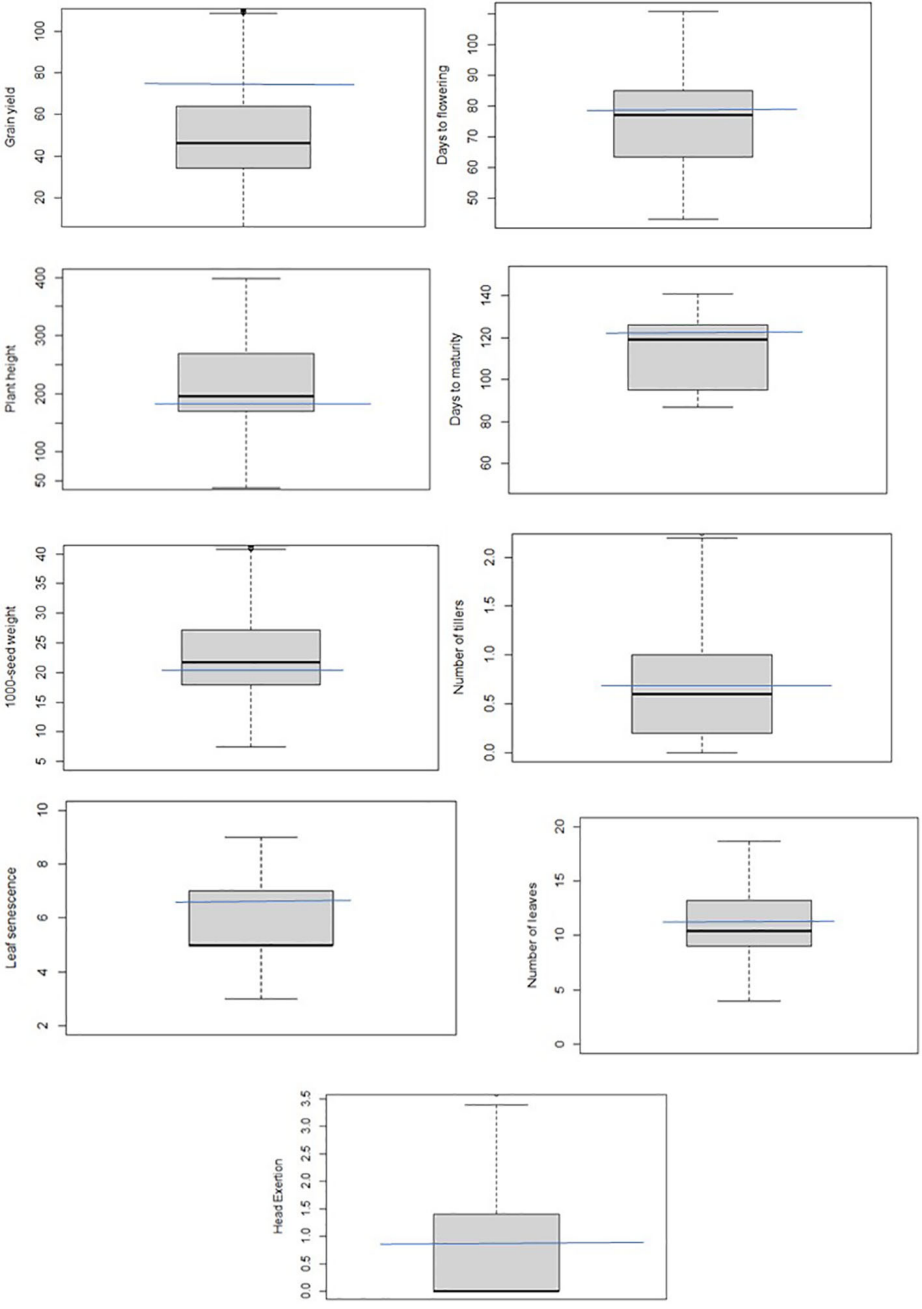
Box plots showing the phenotypic distribution of the studied traits in the BCNAM population or an illustration of the variation of values in the dataset. The blue line passing through it indicates the position of the recurrent parent (Teshale) in the population.

**Figure 3:**
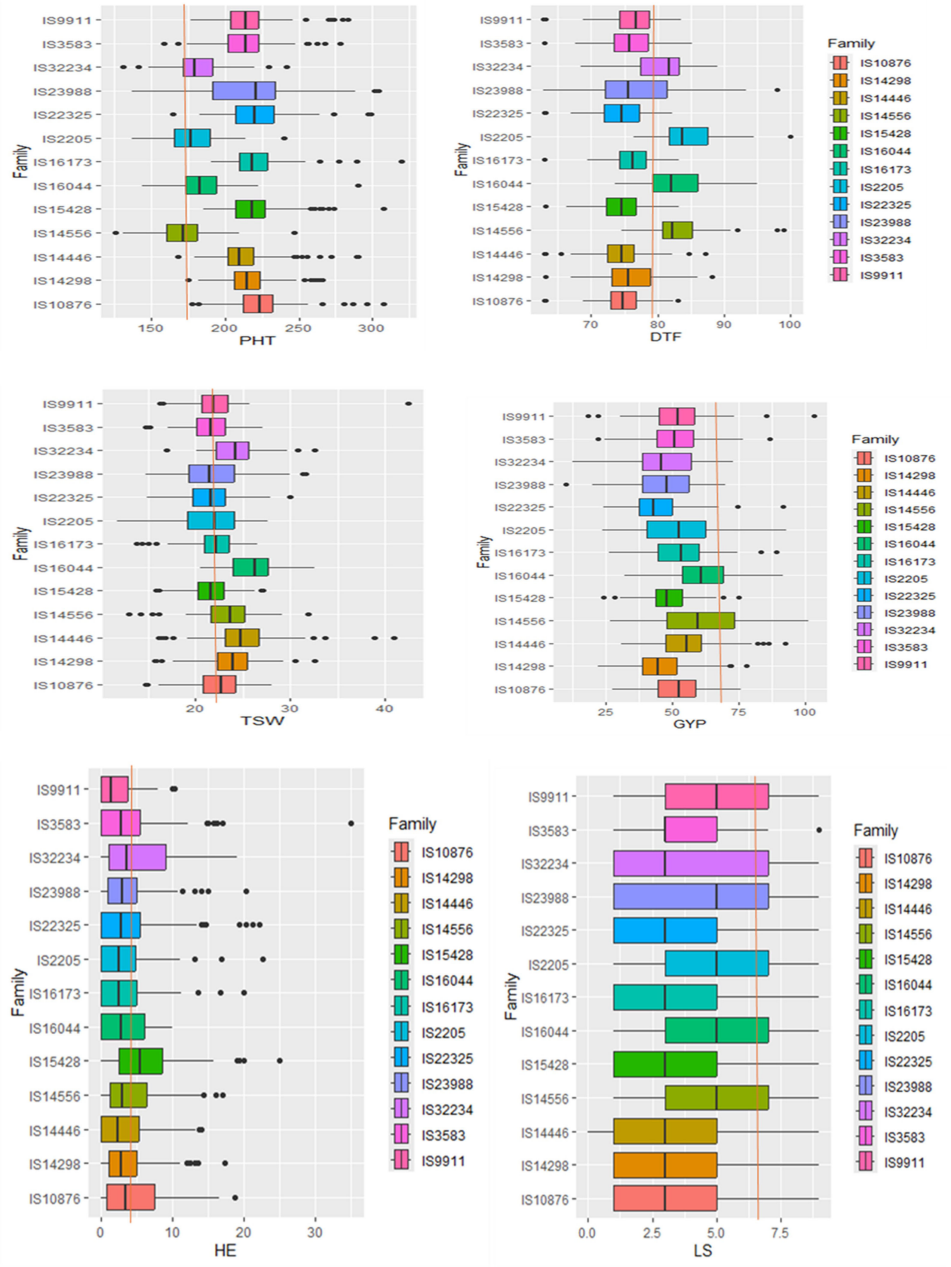
A box plot showing the distribution of six key traits for each family. DTF, the number of days till 50% flowering; PHT, plant height, GYP, grain yield per plant; TSW, 1,000-seed weight; LS, leaf senescence; HE, head exertion. The red line passing through the box plots illustrates the position of the recurrent parent Teshale in relation to the other genotypes arising from each distinct population.

Plant height, grain yield, 1,000-seed weight, and days to flowering were more variable than other traits, whereas variation for number of leaves, number of tillers, leaf senescence, and days to maturity were quite limited in this population (**Figure 2**). Overall, the grand mean across all populations was close to the values of Teshale for all traits except grain yield and leaf senescence. Grand means for grain yield and leaf senescence were much below Teshale’s. Exotic donor parents also exhibited significant variation for traits such as grain yield, plant height, days to flowering, and 1,000-seed weight. The highest variation among progenies for grain yield was recorded from parents such as IS14556, IS2205, IS16044, and IS32234 (**Figure 3**). IS22025, IS23988, and IS4446 also displayed the highest family variation for 1,000-seed weight.

Most traits exhibited similar phenotypic distributions in terms of population mean and standard deviation for most of the populations. Individual families had produced progenies with phenotypic performance comparable to those of Teshale, the recurrent parent (**Figure 3**). The progeny values of individual families of the 13 donor parents’ grain yield, for instance, varied from 10.17 (IS23988) to 103.30 (IS9911), and for 1,000-seed weight, they varied from 4 (IS2205) to 46 (IS9911). Teshale’s values fell near the middle of these ranges, at 72.00 and 22.67, respectively. Other traits also showed a similar pattern consistent with the backcross breeding strategy. The family boxplot showed that IS16044, IS2205, and IS14556 produced many interesting variants for grain yield, 1,000-seed weight, days to flowering, and plant height.

Broad sense heritability estimates ranged from 6.6 to 96.42 (**Supplementary Table 1**). It was relatively higher for DTF, DTM, PHT, LS, HE, GYP, and 1,000-seed weight. Genetic advance as percent of mean (GAM) was relatively higher for days to flowering followed by leaf senescence, days to maturity, head exertion, and grain yield. It was low for plant height, number of leaves, number of tillers, and 1,000-seed weight. Relatively high heritability coupled with high GCV and genetic advance over mean was observed for days to flowering, leaf senescence, and head exertion. Overall, the BCNAM population exhibited considerable variation and heritability for various traits demonstrating that a diverse array of plant types was preserved from short (line 1356) to tall height (line 1390), and from high (line 1180) to low (line 1278) yield.

### Correlation among traits

**Figure 4:**
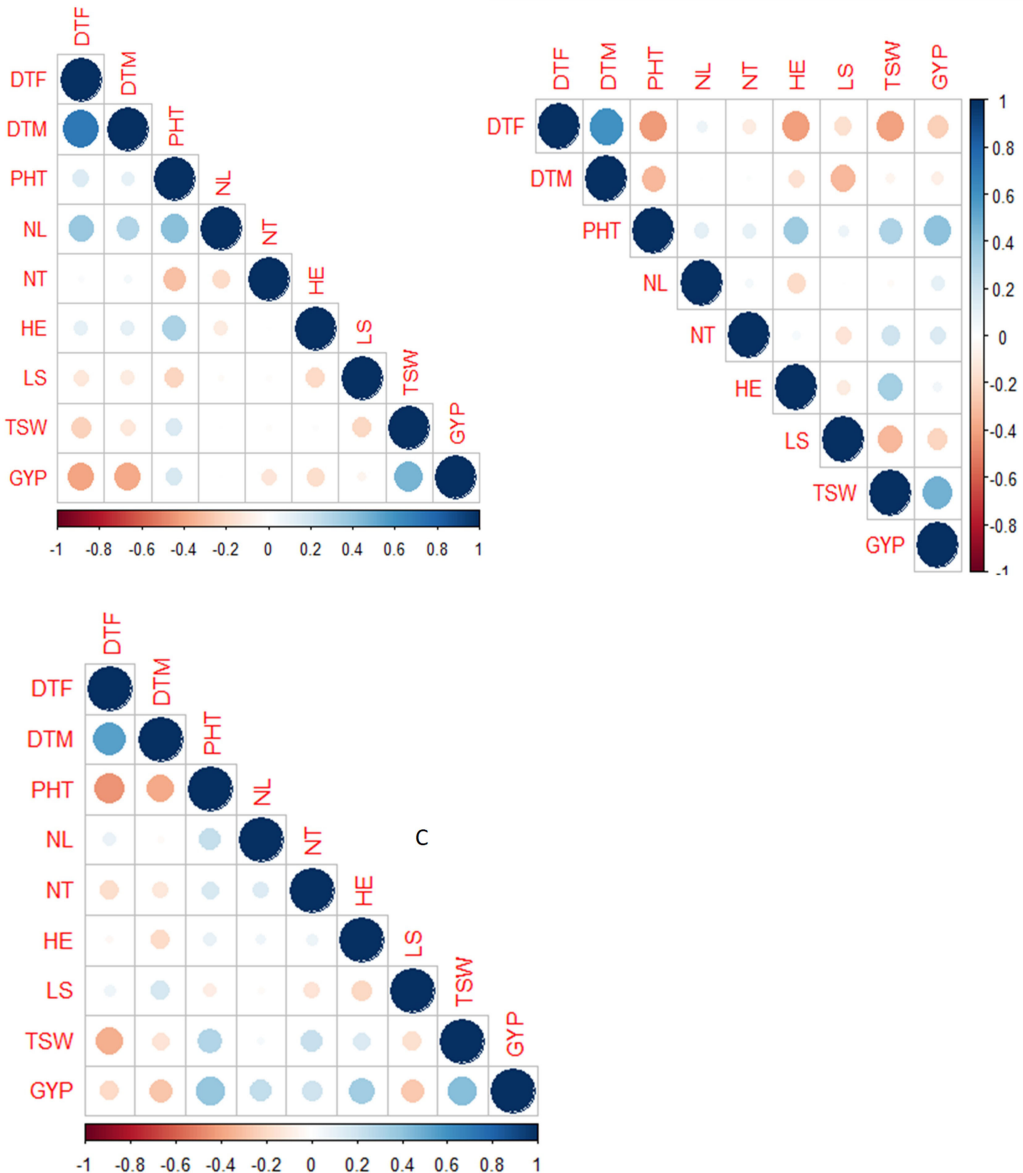
Correlations of nine traits studied in the BCNAM population for the three study sites (A = Kobo, B = Meiso, and C = Sheraro). DTF, days to 50% flowering; DTM, days to maturity; NL, number of leaves; HE, head exertion; LS, leaf senescence; NT, number of tillers; PHT, plant height; GYP, grain yield per plant; TSW, 1,000-seed weight.

Grain yield expressed strong and positive correlation with PHT (r = 0.40 at Kobo, 0.22 at Meiso, 0.40 at Sheraro) and TSW (r = 0.40 at Kobo, 0.45 at Meiso, and 0.45 at Sheraro) at all three locations (**Figure 4**). However, it was negatively and significantly correlated with days to flowering (r = −0.43 at Kobo, −0.25 at Meiso, and −0.2 at Sheraro), days to maturity (r = −0.45 at Kobo, −0.13 at Meiso, and −0.35 at Sheraro), and leaf senescence (not significant at Kobo, −0.25 at Meiso, and −0.35 at Sheraro) at all three locations. There were also low-to-moderate negative correlations between the 1,000-seed weight and both days to flowering and days to maturity. In general, there are notable associations between grain yield and plant height, 1,000-seed weight, head exertion, days to flowering, and maturity in all three locations suggesting that these traits could be the primary factors driving yield under moisture stress conditions.

**Figure 5:**
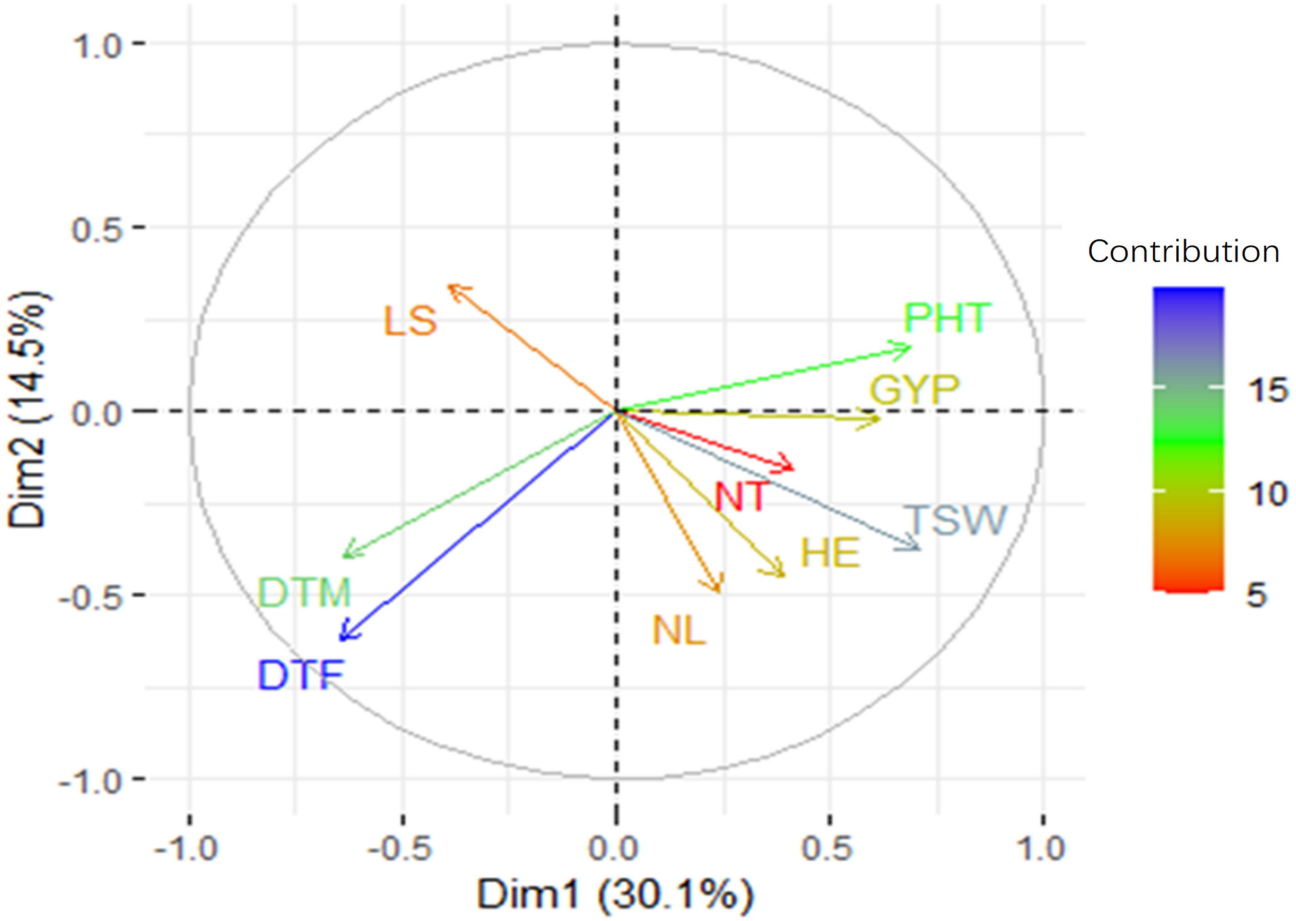
A PCA plot with the loading values for each attribute and the correlation between them based on a two-dimensional regression analysis. DTF, days to 50% flowering; DTM, days to maturity; NL, number of leaves; HE, head exertion; LS, leaf senescence; NT, number of tillers; PHT, plant height; GYP, grain yield per plant; TSW, 1,000-seed weight.

To validate the simple linear regression analysis further, we performed PCA analysis (**Figure 5**). PC1 accounted for 30.1% of the variance in the data, whereas PC2 explained 14.5%. The most contributing traits in each component were DTF and DTM in PC2, and PHT, TSW, DTF, DTM, and GYP in PC1. The relationships between traits deviated only modestly from single linear regression analysis further supporting that plant height, 1,000-seed weight, days to flowering, and maturity could serve as criteria for selecting genotypes under moisture stress condition in this case. Given the intricate inheritance and genetic linkage of quantitative characteristics, some trade-offs are expected.

### Identification of favorable progenies for grain yield and other traits

The individual genotype means for the traits measured from the BCNAM populations are summarized in **Figure 3**, **Table 3**, **Supplementary Table 2**. Considerable differences in mean values were observed among the BCNAM lines with respect to all the traits studied. Donor parent IS14556 produced the cross with the best mean performance among the 13 parents in terms of grain yield. Twelve lines were found to have higher GYP than the better parent. While progenies that performed significantly better than the recurrent parent were infrequent in the other populations, every population had at least one, and four populations (IS14446, IS14556, IS16044, IS10876) had more than the 5% that could be explained by chance. The highest average grain yield was recorded from 1180 (103.36 g, IS9911), 1373 (101.23 g, IS14556), 1318 (92.60 g, IS2205), and 1 (92.55 g, IS14446) lines.

**Table 3:** Combined means for five traits of the top 15 best and bottom performing genotypes, check and recurrent parents of the BCNAM population ranked according to their performance during the 2018 cropping season.

Lowest values											
Crosses	Gen	DTF	Crosses	Gen	DTM	Crosses	Gen	PHT	Crosses	Gen	GYP
IS9911	1199	62.83	IS14446	20	87.00	IS14556	1356	126.00	IS23988	1278	10.17
IS23988	1263	62.84	IS14446	22	87.00	IS14556	1378	130.70	IS32234	1462	12.38
IS3583	1101	62.88	IS14446	86	87.00	IS32234	1463	130.80	IS9911	1206	18.20
IS9911	1204	62.89	IS10876	216	87.00	IS23988	1263	136.80	IS23988	1270	20.02
IS23988	1255	62.90	IS10876	330	87.00	IS2205	1289	137.30	IS32234	1438	21.37
IS14446	135	62.91	IS22325	560	87.00	IS32234	1461	141.00	IS3583	1084	21.94
IS22325	617	62.92	IS22325	617	87.00	IS32234	1462	141.00	IS23988	1260	22.00
IS16173	839	62.94	IS16173	839	87.00	IS16044	1398	144.10	IS9911	1205	22.13
IS22325	550	62.98	IS3583	1101	87.00	IS23988	1277	145.00	IS14298	811	22.28
IS16173	859	62.98	IS23988	1214	87.00	IS32234	1464	148.00	IS23988	1232	22.98
IS10876	201	62.99	IS23988	1255	87.00	IS2205	1300	148.20	IS32234	1437	23.14
IS23988	1222	63.00	IS23988	1259	87.00	IS2205	1296	148.30	IS2205	1308	23.66
IS23988	1259	63.01	IS9911	1199	87.00	IS14556	1344	150.00	IS15428	399	24.08
IS10876	191	63.02	IS22325	550	91.00	IS14556	1351	150.60	IS22325	560	24.40
IS22325	560	63.02	IS16173	859	91.00	IS16044	1409	150.70	IS3583	1101	24.80
Highest values											
IS14556	1370	91.00	IS14556	1376	129.80	IS23988	1239	288.50	IS9911	1184	85.25
IS16044	1381	91.00	IS23988	1270	130.00	IS14446	86	289.40	IS16044	1397	85.48
IS16044	1403	91.00	IS2205	1318	130.00	IS14446	135	289.80	IS14446	4	86.09
IS16044	1426	91.00	IS2205	1320	130.20	IS10876	330	296.60	IS3583	970	86.39
IS14556	1378	92.00	IS23988	1276	130.70	IS22325	550	296.90	IS16044	1421	86.53
IS2205	1329	92.33	IS2205	1322	130.70	IS22325	626	299.30	IS14556	1366	86.81
IS2205	1320	93.25	IS2205	1324	131.30	IS23988	1222	301.20	IS14556	1374	88.66
IS23988	1270	93.33	IS23988	1278	132.00	IS23988	1258	304.30	IS16173	905	88.91
IS2205	1321	94.50	IS2205	1321	132.50	IS10876	191	307.50	IS14556	1357	89.41
IS16044	1393	95.00	IS32234	1437	132.70	IS15428	501	307.50	IS22325	660	91.50
IS23988	1277	98.00	IS32234	1435	133.00	IS14446	138	320.70	IS16044	1419	91.56
IS23988	1278	98.00	IS2205	1315	133.30	IS14446	52	327.30	IS14446	1	92.55
IS14556	1368	98.00	IS2205	1317	133.50	IS16173	859	330.00	IS2205	1318	92.60
IS14556	1379	99.00	IS23988	1277	137.00	IS16173	960	332.00	IS14556	1373	101.23
IS2205	1288	100.00	IS14556	1379	141.00	IS16044	1390	334.00	IS9911	1180	103.36
Parents and check											
IS14446	1466	82.00	IS14446	1466	137.70	IS14446	1466	151.50	IS14446	1466	43.40
IS10876	1467	90.25	IS10876	1467	123.20	IS10876	1467	208.20	IS10876	1467	60.20
IS15428	1468	92.50	IS15428	1468	129.00	IS15428	1468	159.50	IS15428	1468	70.42
IS22325	1469	75.25	IS22325	1469	131.80	IS22325	1469	216.60	IS22325	1469	58.91
IS14298	1470	83.50	IS14298	1470	127.80	IS14298	1470	200.20	IS14298	1470	51.31
IS16173	1471	78.00	IS16173	1471	120.20	IS16173	1471	180.80	IS16173	1471	71.79
IS3583	1472	78.00	IS3582	1472	118.70	IS3582	1472	138.80	IS3582	1472	65.14
IS9911	1473	85.00	IS9911	1473	138.00	IS9911	1473	135.30	IS9911	1473	51.36
IS23988	1474	85.67	IS23988	1474	128.70	IS23988	1474	221.80	IS23988	1474	49.05
IS2205	1475	95.00	IS2205	1475	141.00	IS2205	1475	243-00	IS2205	1475	32.74
IS14556	1476	73.00	IS14556	1476	131.00	IS14556	1476	218.80	IS14556	1476	60.00
IS16044	1477	79.50	IS16044	1477	122.80	IS16044	1477	213.80	IS16044	1477	71.86
IS32234	1478	70.00	IS32234	1478	117.00	IS32234	1478	201.50	IS32234	1478	16.57
Teshale	1479	79.50	Teshale	1479	121.00	Teshale	1479	174.40	Teshale	1479	72.00
Melkam		78.00	Melkam		105.00	Melkam		148.20	Melkam		75.10
Mean		80.38	Mean		123.98	Mean		179.25	Mean		61.49
CV		4.54	CV		7.64	CV		33.50	CV		18.50
LSD (0.05)		7.60	LSD (0.05)		10.22	LSD _(0.05)_		16.45	LSD_(0.05)_		18.53

DTF, days to flowering; DTM, days to maturity; Gen, genotype ID; LSD, least significant difference; CV, coefficients of variations; PHT, plant height; GYP, grain yield per plant.

Lines, such as 1182 (IS9911), 67 (IS14446), 684 (IS14298), and 1210 (IS32234), registered superior mean performance for the 1,000-seed weight over the recurrent parent. The average 1,000-seed weight was 26.49 g. The lowest 1,000-seed weight value was from line 1334 (IS2205), followed by lines 1372 (IS14556), 839 (IS16173), and 1378 (IS14556). The lines with the highest 1,000-seed weight were those descended from donor parents IS9911, IS14446, IS14298, and IS23988.

The majority of families had mean head exertion values that were higher than the recurrent parent (**Figure 3**). Overall, the mean values across all families were close to the values of Teshale for all families except IS9911, which was much lower. In addition, we assessed leaf senescence on an ordinal scale of 1 (least senescent) to 9 (most or completely senescent leaf). Compared to Teshale (6.10), the majority of families displayed lower leaf senescence with population means ranging from 1.00 in IS14446 to 8.45 in IS3583. Leaf senescence and head exertion were also significantly correlated with grain yield and had a notable negative relationship with one another in all the three locations.

Line 1199 (from donor parent IS9911) followed by 1263 (62.84 days from family IS23988) had shorter flowering (62.83 days) than others, which is more suitable to moisture-stressed areas, as the earliness traits enable early maturity and escape of the stress period. The majority of these lines displayed a shorter flowering period than the recurrent parent, Teshale. All 13 populations showed evidence of transgressive segregants for DTF, with the overwhelming majority being early flowering in nine populations (**Supplementary Table 2**) and with 37 days difference between the early and late flowering genotypes. Most of the lines matured within a 54-day window (87–141 days) with an average of 123.98 days. Lines, such as 20 (87 days, IS14446), 22 (87 days, IS14446), 86 (87 days, IS14446), and 216 (87 days, IS10876) had maturities earlier than the recurrent parent. Most of these early maturing lines were obtained from progenies of IS14446 and IS10876 parents. Patterns of transgression for days to maturity closely mimicked those of days to flowering.

The means for plant height of the majority of the populations were between 126 and 334 cm. Curiously, the populations that tended to produce abundant transgressants for early flowering also tended to produce larger numbers of transgressants that were taller than the tall parent. The highest plant height was obtained from lines 1390 (334 cm, IS16044), 960 (332 cm, IS16173), and 859 (330 cm, IS16173). The difference in height from the other genotypes was significant. The shortest plant height was recorded from lines 1356 (126 cm, IS14556), 1378 (130.70 cm, IS14556), and 1463 (130.80 cm, IS32234).

### Transpiration efficiency and H_2_O extraction contributions in the progenies

**Figure 6:**
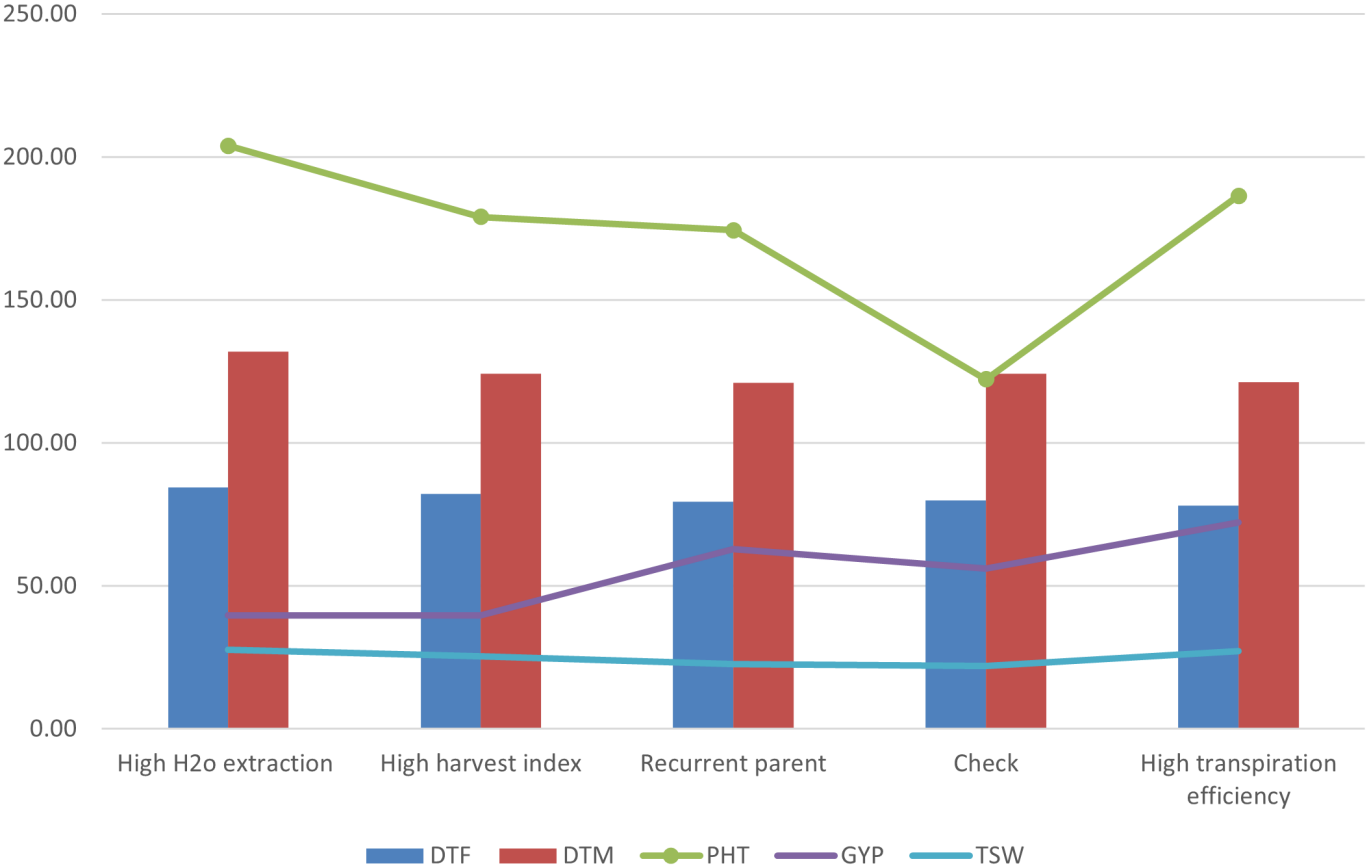
The average progenies performance for GYP, DTM, DTF, PHT, and TSW based on background features such as transpiration efficiency, water extraction, and harvest index.

**Figure 7:**
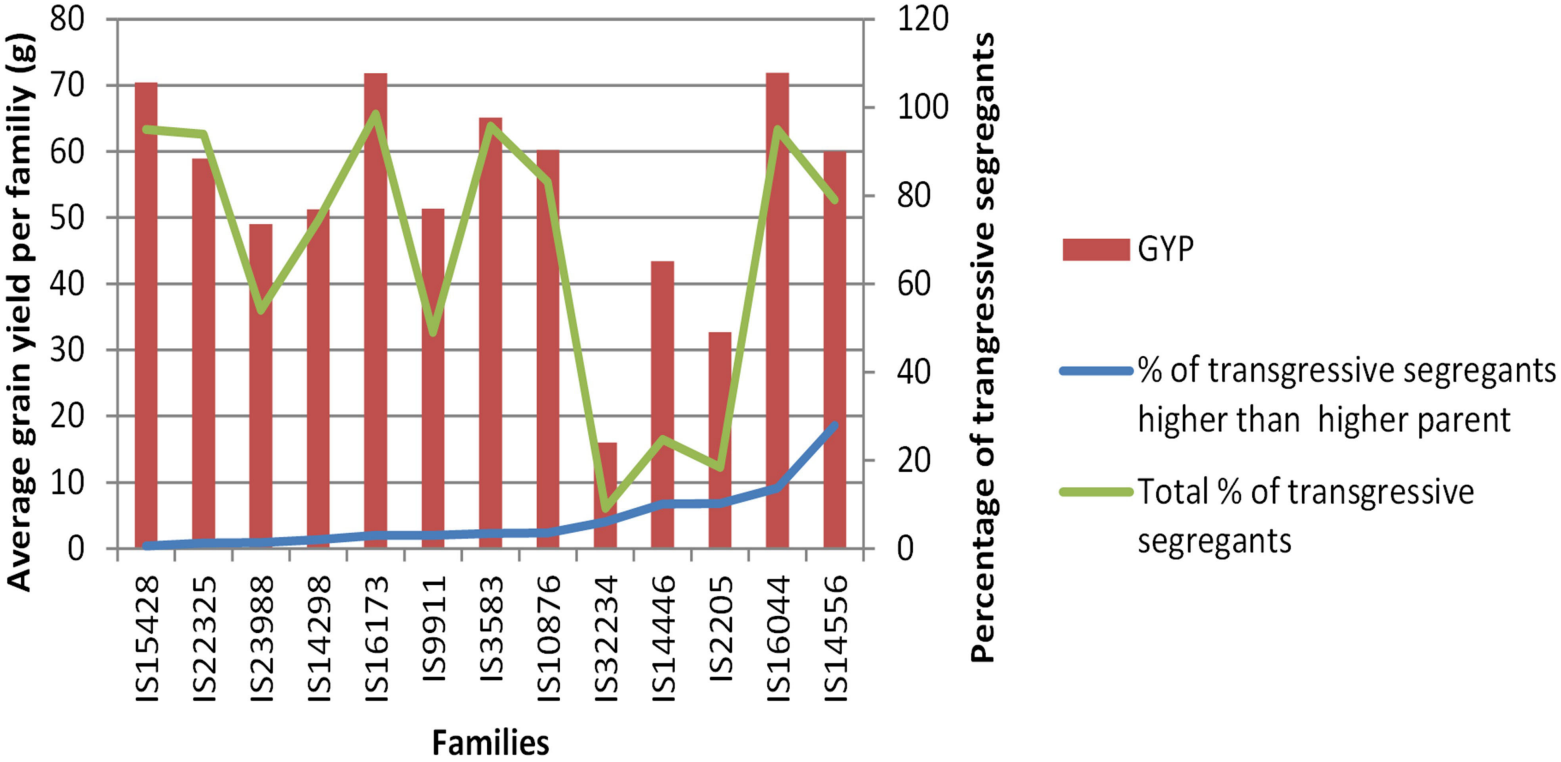
Average grain yields per family and percentage of transgressive segregants per family in the BCNAM populations.

Our exotic donor parents included three with high H_2_O extraction, three with high harvest index, and the remaining seven with high transpiration efficiency. The average of progenies obtained from the three donor parents with high H_2_O extraction background showed lower grain yield, late maturity, delayed flowering, and low 1,000-seed weight compared to the recurrent parent (**Figure 6**). The progenies derived from the average of four donor parents with high transpiration efficiency, on the other hand, showed high grain yield as well as relatively early flowering and maturity, as was also reflected in the parents. The high harvest index parent recorded modest yield, flowering, and maturity time. For the adaptation-related traits, in general, the donor parents with high transpiration efficiency displayed promising performance that could be attributed to the genetic makeup for the parents. Overall, donor parents IS14556 and IS16044 seemed effective in conferring drought stress tolerance-related characters. This could be reflected through the high average performance of lines from such donors and higher frequencies of transgressants (**Figures 3**, **7**). In the figure, it is clearly observed that the two exotic parents outperformed the other parents and gave comparable yield to the recurrent parent.

## Discussion

### BCNAM as a novel source of genetic variation for sorghum improvement

Overall, this study demonstrated a wide range of variability among progenies for most of the characters tested indicating their potential for use in future sorghum improvement. The study discovered adequate variation for drought resistance and demonstrated the viability of selecting favorable genotypes based on traits that are highly associated with yield under moisture stress conditions. The germplasm presented in this research incorporates favorable alleles from numerous exotic parental lines into relevant genetic backgrounds that combine suitable yield and quality with drought resilience components. The variety of drought-responsive properties in donor parents, including transpiration efficiency, water extraction, and harvest index traits (Vadez et al., 2011a, b), may play an essential role in the recombination and formation of such an important population.

Plant tolerance to drought stress is a complicated trait, and identifying suitable genotypes based on stress exposure under natural circumstances or sequentially occurring stress in greenhouse conditions is challenging (Vadez et al., 2024). Moreover, different traits can be used to screen genotypes and evaluate stress tolerance. For instance, early flowering or maturity is commonly seen as a drought-escape strategy, and reduced flowering delay under drought has been proposed as an indicator of drought tolerance in some environments (Monkham et al., 2015). Leaf senescence and head exertion have been also targeted for phenotyping drought response in sorghum (Harris et al., 2007).

Grain yield is a complex variable that is heavily impacted by the environment. Correlation coefficients, as indicators of the degree of relationship between different attributes, are useful in determining those characters that are highly associated with grain yield and, consequently, can be used as indicators in selection for yield. In our study, we included plant height, 1,000-seed weight, head exertion, leaf senescence, days to flowering, and maturity, all of which were found to be substantially correlated with grain yield in a two-stage analysis. The significant correlation observed between grain yield and those traits suggests that these traits may be the primary factors driving yield under moisture stress conditions, though drought adaptation traits can have either positive or negative effects on crop performance depending on the drought scenario (Vadez et al., 2024). Indeed, we noticed the prevalence of certain traits in certain environments, like earliness (flowering and maturity time) in Kobo, while traits that reflected plant vigor potential (plant height) and capacity to fill grains (thousand seed weight, leaf senescence, head exertion) were more important at Meiso and Sheraro. A benefit of the BCNAM approach was in providing a variety of segregants for each of these traits, which could be used to develop drought-tolerant cultivars suitable for each of the representative drought scenarios.

Higher variation was observed for flowering, plant height, grain yield, and 1,000-seed weight in the BCNAM population. However, in the present study, limited variation was obtained for tiller number and days to maturity in the BCNAM population. This might be because all biparental populations in this BCNAM involved two *S. bicolor* parents, which exhibited no significant differences in tiller morphology. Substantial phenotypic variation for tillering has been reported previously in *Sorghum halepense* (Paterson et al., 1995; Feltus et al., 2006), a natural interspecific hybrid between *S. bicolor* and its weedy relative *S. propinquum*.

The presence of high heritability coupled with high genetic advance as percent of the mean (GAM) for some traits would also give added options for improvement of traits through selection. For characters, such as DTF, HE, and PHT with relatively high heritability (‗60), selection could be effective. Those traits might be regulated by additive gene action, and phenotypic selection could be effective as additive gene action governs the fixable component of variation in breeding (Singh et al., 2001).

### Promising lines are identified for key adaptive traits

Higher variation was observed for flowering and plant height across the BCNAM populations than the other traits and is a prerequisite for sorghum breeders to develop cultivars that can withstand moisture stress. The high variation for those traits in response to moisture stress can be attributed to difference in the diverse set of donor parents that carry a unique set of favorable alleles for the target regions. Similar findings were made by Mace et al. (2013) and Jordan et al. (2011) who assessed the drought response of sorghum NAM populations and reported significant variation among progenies for flowering and maturity time. Chahota et al. (2007) reported early flowering and maturing progenies from an F3 sorghum segregating populations, partially in agreement with the present study.

To develop cultivars suitable for diverse climates, control of flowering time becomes of central importance to sorghum breeding programs. Determining the variation of flowering genes remains one key to better understanding the evolution and genomic diversification patterns in sorghum. Most of the populations in the present study included progeny with both earlier and later flowering than the recurrent parent demonstrating the presence of transgressive segregants in both directions. Lines 1199, 1263, 1101, and 1204 had relatively earlier flowering than others making them suitable for drought escape. Early flowering is one of the most desirable drought tolerance-related attributes as it has major impacts on crop performance under terminal drought, with relatively minor variations in crop duration that could lead to significant differences in grain yield (Hammer, 2006). For example, flowering time alone often explains high percentages of yield difference during drought, such as 50% in pearl millet (Bidinger et al., 1987). Early flowering was a key trait in Kobo.

Most of the lines matured within a 54-day window (87–141 days) with an average of 123.86 days. Lines, such as 20, 22, and 86, had maturities earlier than the recurrent parent making them suitable for drought escape in areas where terminal moisture stress is predominant. Most populations contained individual progenies that were significantly taller or shorter than the recurrent parent. Reduced moisture availability disrupts plant function and growth resulting in decreased agricultural yields. When stress gets severe, sorghum plants tend to transition from the vegetative to the reproductive phase resulting in short stature (Barnabás et al., 2008). As a result, this population with the highest variation in plant height would help in the identification of appropriate genotypes for the region.

### Promising lines are identified for traits that reflect better grain filling

Genotypes, such as 1180, 1373, 1318, and 1, displayed higher average grain yield than the recurrent parent over the three environments signifying their worth for further moisture stress adaptation breeding programs, as the average performance under moisture stress is one of the selection criteria to get superior lines for drought tolerance (Blum, 2011). Several studies have examined the effects of drought stress on sorghum yield. For example, Assefa et al. (2010) found that drought stress during the vegetative and reproductive phases lowered sorghum output by more than 36% and 55%, respectively. Abraha et al. (2015) also reported the effects of moisture stress on sorghum growth and development resulting in a considerable loss of grain yield.

One critical feature of this research is proving the relationship between grain yield, LS, HE, and TSW under drought stress circumstances. Grain yield and 1,000-seed weight exhibited substantial variability in the population and had a strong relationship across all three conditions. Based on family performance, IS9911 and IS14556 produced the maximum grain output and 1,000-seed weight. In general, based on multiple metrics, individual families demonstrated better resistance to drought than the recurrent parent did. First, as an indicator of drought tolerance (Feltus et al., 2006; Harris et al., 2007), the majority of families exhibited higher mean head exertion values and lower leaf senescence than the recurrent parent. Furthermore, there are an adequate number of progenies that produce higher yield, but earlier flowering than the recurrent parent is a favorable trait for drought-prone locations where terminal moisture stress is common. Likewise, the presence of high-performing genotypes (Jordan et al., 2011) and the reliability of plant height, grain yield, and 1,000-seed weight as selection criteria have been reported in sorghum (Techale et al., 2022; Kebede et al., 2001; Chahota et al., 2007).

### Transpiration efficiency (TE) contributed for high yield

Transpiration efficiency at the plant level (TE), defined as the total dry matter generated per unit of water transpired, is an important agricultural attribute, particularly as water resources become scarcer in many areas. In the present study, the donor parents with high transpiration efficiency (TE) displayed promising performance for yield and adaptation-related traits, though *de novo* data were not collected on this specific trait relying on prior characterization of the donor lines by Vadez et al. (2011a, b). Those parents registered early flowering and maturity, which made them good candidates for drought escape. The observed association between TE and adaptive traits could be attributed to an intrinsic fast allocation of assimilates to shoots under limited water availability. Several studies have demonstrated connections between TE and biomass allocation that illustrate source–sink relationships. Nathan et al. (2021) reported that stressed environments cause most plants to flower earlier, and flowers begin to produce seeds before the effects of stress become fatal, which is one adaptation mechanism in sorghum and other C4 grasses. For example, drought modifies the timing of flowering in *Mimulus guttatus* (Nicholas et al., 2014) and causes *Brassica rapa* to bloom earlier (Franks et al., 2007). The benefit of high TE alleles from the donor parents was also akin to providing an extra supply of water that could have been used during grain filling.

The average of progenies from donor parents with high transpiration efficiency displayed high yield. According to Vadez et al. (2011), TE and water extraction are not mutually exclusive and were the main drivers of yield differences in sorghum during terminal drought, which agrees with the present findings. Yield (Y) is determined by the amount of water extracted from soil for transpiration (T), the conversion of transpiration water into biomass (TE), and the conversion of biomass into grains via the harvest index (HI) (Y = T × TE × HI) (Kenton et al., 2020). Hence, in such environments, the use of those donor parents and breeding for high drought stress adaptation seemed to be effective.

In general, the crossing described here appeared effective in transferring beneficial alleles contributing to drought tolerance and producing genotypes with high yield. This could be attributed to extensive shuffling of genetic variation during population development that would lead to formation of novel allelic combinations (Huynh et al., 2018). In particular, IS14556 and IS16044 were effective in conferring drought stress tolerance-related characters, as reflected through high average performance of lines from these donors. The higher average grain yield for these families could be due to increased frequency of desirable alleles for grain yield and other contributing traits in the donor parents that boosted the performance of the BC1F4 lines. This highlights that improvement in water-limited areas will likely profit more from making use of these genetic resources and exploiting traits such as head exertion, leaf senescence, 1,000-seed weight, plant height, and flowering time.

## Conclusions and recommendations

In the present study, the BCNAM approach has been demonstrated to be an effective method to transfer beneficial alleles/characters from exotic sorghum germplasm into an Ethiopian elite genetic background. It produced many transgressive segregants that appear, based on early testing, to perform better than an existing variety under moisture stress conditions. Lines with significantly higher grain yield components were identified that would potentially adapt to a range of moisture stress environments. Likewise, those lines with the shortest average days to flowering and days to maturity could be used as potential candidate genetic materials for drought escape research across a range of environments where terminal moisture stress is a problem. Therefore, those lines that had higher grain yield and short flowering time appear well suited for further drought stress adaptation breeding research across a range of environments.

## Data Availability

The original contributions presented in the study are included in the article/**Supplementary Material**. Further inquiries can be directed to the corresponding author.
