# Supplementary material for: Harnessing the genetic potential of exotic sorghum germplasm for drought resilience in arid regions of Ethiopia
Source: Front Plant Sci. 2025 Apr 9;16:1548591. doi: 10.3389/fpls.2025.1548591 (PMC12014701; doi:10.3389/fpls.2025.1548591)
Supplement: Supplementary file 1 [file Table1.docx]

**Supplemental Table 1** Estimates of genetic parameters for nine traits among 1480 sorghum BC1F4 genotypes grown at Kobo, Sheraro and Meiso during the 2018 cropping season

| Traits | Mean+ SD | Range | σ^2^p | σ^2^g | PCV | GCV | h^2^b | GA | GAM |
| --- | --- | --- | --- | --- | --- | --- | --- | --- | --- |
| DTF | 80.83+4.80 | 62.83-100.00 | 57.51 | 55.45 | 9.38 | 9.21 | 96.42 | 19.01 | 23.52 |
| DTM | 123.98+3.50 | 87.00-141.00 | 66.86 | 53.37 | 6.60 | 5.89 | 79.83 | 16.97 | 13.69 |
| NT | 0.80+1.0 | 0.40-3.25 | 6.63 | 1.87 | 5.92 | 3.14 | 28.13 | 1.88 | 4.33 |
| PHT | 179.25+25.90 | 126.0-334.00 | 71.37 | 37.91 | 4.71 | 3.44 | 53.13 | 11.67 | 6.51 |
| HE | 5.71+4.50 | 0.00-31.25 | 18.68 | 14.45 | 75.69 | 66.57 | 77.37 | 8.69 | 152.25 |
| GYP | 61.49+16.50 | 10.17-103.36 | 46.67 | 28.58 | 11.11 | 8.69 | 61.24 | 10.88 | 17.69 |
| TSW | 26.49+2.30 | 11.76-55.33 | 6.60 | 1.77 | 9.70 | 5.02 | 26.80 | 1.79 | 6.76 |
| LS | 4.52+ 1.23 | 2.00-9.00 | 2.10 | 0.89 | 32.10 | 25.84 | 64.78 | 931.23 | 42.09 |
| NL | 10.95 + 0.95 | 8.95-13.15 | 1.82 | 0.11 | 12.37 | 3.19 | 6.66 | 15.52 | 1.70 |

DTF= days to flowering, DTM= days to maturity, NT= number of tillers, PHT=plant height, HE=panicle exertion, GYP=grain yield per plant, TSW= thousand seed weight, NL= number of leaves per plant, LS= leaf senescence, SD=standard deviation, σ^2^g=genotypic variance, σ^2^p=phenotypic variance, h^2^b=Broad sense heritability,

**Supplemental Table 2** Lowest and highest values of the BCNAM populations, parents and number of transgressive segregants for four traits of the BC1F4 sorghum populations

|  | BC1F4 generation | | | Parents | | No. of transgressive segregants | |
| --- | --- | --- | --- | --- | --- | --- | --- |
| Populations | Lowest  Value | | Highest  Value | Lower  value | Higher  Value | Lower than  lowest parent | Higher than  Highest  parent |
| **Days to flowering** | | | | | | | |
| IS14446 | | 62.50 | 87.24 | 79.50 | 82.00 | 169.00 | 3.00 |
| IS10876 | | 62.90 | 83.00 | 78.25 | 79.50 | 154.00 | 8.00 |
| IS15428 | | 63.03 | 83.12 | 79.12 | 79.50 | 157.00 | 12.00 |
| IS22325 | | 62.29 | 82.21 | 75.25 | 79.50 | 95.00 | 10.00 |
| IS14298 | | 63.03 | 88.14 | 79.50 | 83.50 | 113.00 | 6.00 |
| IS16173 | | 70.23 | 83.23 | 78.00 | 79.50 | 96.00 | 22.00 |
| IS3583 | | 67.19 | 85.25 | 79.50 | 89.00 | 76.00 | 0.00 |
| IS9911 | | 62.83 | 83.50 | 79.50 | 85.00 | 82.00 | 0.00 |
| IS23988 | | 69.00 | 98.00 | 79.50 | 85.67 | 49.00 | 8.00 |
| IS2205 | | 76.33 | 100.00 | 79.50 | 89.00 | 6.00 | 7.00 |
| IS14556 | | 74.56 | 99.00 | 73.00 | 79.55 | 2.00 | 32.00 |
| IS16044 | | 73.50 | 95.00 | 79.50 | 79.50 | 17.00 | 33.00 |
| IS32234 | | 68.50 | 89.00 | 78.00 | 79.50 | 9.00 | 19.00 |
| **Days to maturity** | | | | | | | |
| IS14446 | | 87.00 | 122.60 | 121.00 | 127.00 | 177.00 | 1.00 |
| IS10876 | | 87.00 | 117.30 | 121.00 | 123.20 | 171.00 | 1.00 |
| IS15428 | | 92.50 | 119.20 | 121.00 | 122.00 | 169.00 | 3.00 |
| IS22325 | | 87.00 | 118.00 | 121.00 | 121.80 | 164.00 | 0.00 |
| IS14298 | | 92.50 | 119.80 | 121.00 | 127.80 | 149.00 | 0.00 |
| IS16173 | | 87.00 | 118.50 | 120.20 | 121.00 | 134.00 | 0.00 |
| IS3583 | | 97.00 | 118.20 | 114.00 | 121.00 | 114.00 | 0.00 |
| IS9911 | | 92.50 | 119.50 | 121.00 | 128.00 | 100.00 | 0.00 |
| IS23988 | | 87.00 | 137.00 | 121.00 | 128.70 | 57.00 | 5.00 |
| IS2205 | | 118.50 | 133.50 | 121.00 | 141.00 | 4.00 | 1.00 |
| IS14556 | | 119.00 | 141.00 | 121.00 | 131.00 | 11.00 | 2.00 |
| IS16044 | | 122.00 | 128.70 | 121.00 | 122.80 | 8.00 | 32.00 |
| IS32234 | | 116.00 | 133.00 | 117.00 | 121.00 | 2.00 | 23.00 |
| **Plant height** | | | | | | | |
| IS14446 | | 167.90 | 327.95 | 151.50 | 174.40 | 0.00 | 177.00 |
| IS10876 | | 177.90 | 307.52 | 174.40 | 208.20 | 0.00 | 139.00 |
| IS15428 | | 185.20 | 306.50 | 159.50 | 174.40 | 0.00 | 169.00 |
| IS22325 | | 165.10 | 299.30 | 174.40 | 216.60 | 1.00 | 97.00 |
| IS14298 | | 175.50 | 266.00 | 174.40 | 200.20 | 2.00 | 119.00 |
| IS16173 | | 190.70 | 290.20 | 174.40 | 180.80 | 0.00 | 134.00 |
| IS3583 | | 158.40 | 277.70 | 138.80 | 174.40 | 2.00 | 138.00 |
| IS9911 | | 176.90 | 283.00 | 135.30 | 174.40 | 0.00 | 100.00 |
| IS23988 | | 136.80 | 304.40 | 174.40 | 221.80 | 14.00 | 36.00 |
| IS2205 | | 137.30 | 239.70 | 174.40 | 243.00 | 22.00 | 0.00 |
| IS14556 | | 126.00 | 247.10 | 174.40 | 218.80 | 23.00 | 1.00 |
| IS16044 | | 144.10 | 229.80 | 174.40 | 213.80 | 15.00 | 4.00 |
| IS32234 | | 130.80 | 241.26 | 174.40 | 201.51 | 11.00 | 7.00 |
| **Grain yield** | | | | | | | |
| IS14446 | | 31.06 | 92.55 | 43.40 | 72.00 | 26.00 | 11.00 |
| IS10876 | | 27.61 | 75.70 | 60.20 | 72.00 | 136.00 | 6.00 |
| IS15428 | | 24.08 | 74.99 | 70.42 | 72.00 | 168.00 | 1.00 |
| IS22325 | | 26.30 | 91.50 | 58.91 | 72.00 | 152.00 | 2.00 |
| IS14298 | | 22.28 | 77.79 | 51.31 | 72.00 | 108.00 | 3.00 |
| IS16173 | | 26.21 | 88.91 | 71.79 | 72.00 | 128.00 | 4.00 |
| IS3583 | | 21.94 | 86.39 | 65.14 | 72.00 | 132.00 | 5.00 |
| IS9911 | | 18.20 | 103.36 | 51.36 | 72.00 | 46.00 | 3.00 |
| IS23988 | | 10.17 | 70.12 | 49.05 | 72.00 | 39.00 | 0.00 |
| IS2205 | | 23.66 | 92.60 | 32.74 | 72.00 | 4.00 | 5.00 |
| IS14556 | | 26.57 | 101.23 | 60.00 | 72.00 | 22.00 | 12.00 |
| IS16044 | | 32.07 | 91.56 | 71.86 | 72.00 | 44.00 | 7.00 |
| IS32234 | | 12.38 | 72.90 | 16.57 | 72.00 | 1.00 | 2.00 |
